# On the Cremation of the Dead

**Published:** 1874-08-01

**Authors:** 


### ON THE CREMATION OF THE DEAD.

*From La Gazette Medic. de Paris, May* 23, 1874.

MODERN science, in the name of public hygiene, proposes to revive the ancient practice of cremation of the dead, and sets forth excellent reasons for such a cause. These are: the prevention of injury to the living by the inhumation of corpses, and the rendering of soil, air and subterraneous water, as exempt as possible from pestilential germs.

It has been long proven that the terrestrial crust is porous; but the fact is not generally known that earth, sand, dust and pebbles, present interstices of such dimension, that the sum total of air and water which they contain, is almost equivalent to half their volume. This will seem the less surprising, if it be considered that even the densest substances, such as glass and porcelain, are easily penetrated by aqueous and aerial fluids. Thirty years ago, Brongniart discovered that porcelain was an unsuitable material for air thermometers, intended to register furnace temperatures. More recently, Salvetot has reported that enamelled porcelain was penetrable by color solutions, having an aniline composition. Still later, it was observed that the ordinary window glass of a Leipzig hospital, built several years ago, was permeable to water and air. The kitchen of this hospital was located in the basement, and protected against sudden changes of temperature, by strong double windows. The engineer of the establishment, on a warm day in August, carefully cemented the double rows of glass in these windows, which were separated from each other to the extent of about half an inch. To-day the cement has produced such perfect union, that, even with a lens, no interstice can be discovered, and yet water is interposed between the glass plates, to the extent of one-half of their elevation. Whence came it? Assuredly not through the fissures or cracks, for then it would have escaped by evaporation or filtering through the same passages. It is through the pores of the glass, exposed to the kitchen and slightly dilated by the warm air of that apartment, that the water has penetrated. Subsequently it has become condensed by coming into contact with the external parallel glass plates. Precisely such an event occurs upon the walls and ceilings of apartments which are chilled by outdoor air.

If, then, apparently impermeable bodies, such as porcelain and glass, are capable of being traversed by water and air, it is the less surprising that the terrestrial crust should be found to exhibit a greater porosity, and be capable of conducting to the living, by innumerable canals, the deleterious gases emanating from bodies in a stage of decomposition. But, it may be asked, why are those not fatally affected, who, in consequence of the nature of their employment, are compelled to live in a medium which contains putrefying germs? First, it may be responded that, for the most part, they are like the brewers and butchers, who are provided with a free circulation of air in their establishments; and, second, that it is only of late that we have been able to detect the parasites that produce fatal disease, such as those engendered by decomposing substances among the wool-carders and brush manufacturers.

Irrefragable evidence of the mortality induced by cadaveric decomposition, is furnished by the fate of the conquerors of Hannibal. They paid with their lives, under the walls of Syracuse, for the sacrilegious insult offered to the besieged, when they profaned the tombs of their enemies, and scattered their contents over the plains. Quite as conclusive was the typhus endemic of the last century, occasioned by the exhumation of numerous cadavers at Riom, in Auvergne, and it was only thirty years ago that the dead were disinterred, which had been temporarily buried there. Years after, the soil of the ground which had served them for a cemetery, exhaled a disagreeable odor in humid weather. Riecke, in his work *On the Effects of Putrefaction*, to-day a classic, but almost forgotten book, relates a very instructive incident. In the village of Wurtemberg, a common school had been built, as an economic procedure, upon a deserted burial ground. When the winter arrived, the heat of the halls withdrew the air from the soil, and, soon after, masters and scholars being alike stricken down in consequenee of the pestilential emanations, it was found necessary to abandon the situation.

But that which demonstrates more fully than the air, the dangerous character of decomposing substances, is the subterranean water which we drink and bring to the surface by the aid of pumps. Wells are often carelessly dug without taking any precautions, and the water into which the pipe of the instrument is plunged, is consumed without anxiety. Such water may flow from a focus of infection,—perchance from contact with dead animal tissues—no one knows. It is rare that trustworthy information can be had on this point, for the direction of subterranean currents is unknown, except in the rare instances where sub-soil measurements have been made, as to the depth or height of the sea-water, which is to be found everywhere. Now, subterranean water is a vehicle as capable of transporting matter as the water of rivers or rivulets. There are abundant proofs of this. Pettenkofer discovered ammonia in subterranean water forty feet from the gas manufactory, where it was produced. In the last remarkable report of the Faculty of Medicine of Saxe, Reinhard relates that nine large, and several smaller victims of the cattle-plague, were interred at Dresden, at a depth of ten or twelve feet. It was found, the next year, that the water from a well, situate one hundred feet from the pit in which they were buried, had a fetid odor, and contained butyrate of lime. At a distance of twenty feet, it had the disgusting taste of butyric acid, and each quart contained about thirty grains of this substance. The bodies were subsequently disinterred and burned. Foerster, in his studies upon cholera, at Sondershausen, published last year, gives examples of the distance to which impure matter may be transported by subterranean currents. Shortly after the erection of the gas works at that place, well-water, at a distance from them of more than 2,000 feet, had the taste and odor of gas, qualities which were retained until, by repairs, the waste of gas was prevented.

In the face of these results at these distances, of what use are regulations tending to isolate the cities of the dead from the cities of the living? What security is offered by the so-called "protective distance," which is, in Italy, but little more than one hundred yards, and double that in Austria and France? The Hygienic Council, summoned at Brussels in 1852, decided that a distance of four hundred yards was protective, but it has been proven that the radius of danger may extend five hundred feet further. No one can certainly say that putrid material cannot be transported by subterranean water to such a distance, and even, in certain cases, beyond that; and, surely no one could imbibe such water with impunity.

The sole remedy, then, for this evil, is to make sure that the soil contains the fewest possible elements of impurity. This precaution is, above all, essential in cities. In those large centres, where there is a dense population, human industry has, thus far, hardly commenced to learn how to utilize accumulations of fetid material, and public authorities, even with the best of intention, cannot do justice to the claims made upon them. Public hygiene becomes daily more urgent in its demands. It is under the necessity of searching for all methods, by which to satisfy them, and one of these, undoubtedly, is the cremation of dead bodies.

Inhumation and cremation do not essentially differ. In both, the atoms of bodies combine with oxygen and air; in both, the final products of decomposition are carbonic acid, water and ash. But, in the first case, several years and the intervention of other agents are requisite: and here lies the danger to the living, which science must put away.

The technical result to be obtained is the resolution, as speedily as possible, of the organic substance of bodies, into the final and inoffensive products of combustion, and to avoid all intermediate reactions which offend the nostril and injure the health. On the other hand, there are humanitarian considerations of importance. The procedure should produce no disagreeable impressions upon survivors, and should accord with that reverent respect due to the mortal remains of those who were dear to us in life. Let us examine the method by which these ends are to be attained:

Pyre cremation will not suffice: since the burning of the Dresden animals in 1871,—referred to above—which was accomplished under the surveillance of competent authorities, required thirty-six hours in one instance, and twenty-four in another—the materials used having been fagots of wood and bundles of straw saturated with tar. At the close of the operation, the bodies were not completely reduced to cinders, but merely carbonized. This was all that was accomplished. The quantity of wood consumed was not stated.

To burn the corpse of a beloved relative during the hours of an entire day, and then to discover, after no little expense has been incurred, that the remains are only converted into charcoal, cannot be a pleasant experience. The funeral pyre will surely not suffice.

A more acceptable device is suggested by Dr. G. Polli, of Milan—"the gas-pyre." The body, in this arrangement, is introduced into a cylindrical cage made of large wire, placed upon a metal basin, and covered with a thin layer of calcined clay, a portion being spread over the superior surface of the body, and a portion resting upon the plate beneath. Between this envelope and the bars of the cage, two or three parallel circular tubes are disposed horizontally, one above the other. Numberless jets of flame escape from the latter which are supplied with air through apertures made in the edge of the basin. The attendance of the friends of the deceased upon such a cremation, would not be more distressing than at an ordinary burial; but the inventor acknowledges that other senses than that of vision are liable to be affected during the operation, and this fact alone should condemn his procedure.

Brunette, Professor of Anatomy at Padua, prefers the pyre, which he surrounds with walls. The cadaver is then placed upon an apparatus of iron, furnished with two oblong covers of cylindrical shape, which completely enclose the body, and prevent the dispersion of the flames, which are, however, permitted to escape through a longitudinal fenestra made in each cover. According to Brunette, the carbonization of the corpse is accomplished in two hours and a half; it is then reduced to fragments, and submitted to the same action for two additional hours, when white cinders are obtained.

The bones which were exhibited at the Vienna exposition were white, but their fracture was sharp and smooth. From a man weighing ninety pounds, only a little more than four pounds of ash was obtained, after the consumption of 160 pounds of wood. Brunette, therefore, should be credited with having experimentally demonstrated that the cremation of bodies is possible without incurring an inordinate expense. But the actual practice of this operation, and especially the necessity it involves of reducing the carbonized body to fragments, would be repulsive to the feelings of families who might make use of it.

Professor Gorini, of Lodi, operates differently. He first fuses a substance, whose composition is secret, and then immerses the body in the flames of the ignited and boiling liquid. In a work which was published but a few days ago (*Cremation Viewed as a Rational Method, by which to Discharge our Final Duties to the Dead. Zurich: Cæsar Schmidt*), Wegmann Ercolani translates the account of an Italian who witnessed this procedure. We select, for reproduction, the following passage: "Gorini, as soon as the liquid was in ebullition, seized a leg, a foot, a hand, a thigh, and, finally, the head of a human body, extended upon the earth. Each of these parts, as soon as it came into contact with the boiling liquid, burned with a brilliant flame, and, at the end of a brief time, was completely destroyed. The gas and smoke which escaped from the crucible were soon lost in the surrounding atmosphere, and, while the decomposition rapidly progressed, the assistants were unable to detect the slightest odor." This entire mass, therefore, which represented at least the quarter of a cadaver, was burned without noise or odor in an iron crucible, and within a closed laboratory. The account is silent respecting the duration of the cremation and the quantity of the cinders. The results stated, if they be confirmed, indicate that the procedure is well worth recommendation, on account of its simplicity. But while the composition of the liquid employed remains a mystery, it will be impossible to decide as to its merits. We must search for a still different process.

To me, the most satisfactory solution of the problem is the process by *regenerative heat*, to which my attention was first attracted at the universal exposition of 1867, and whose admirable results I have noted in several laboratories. Of all methods, this provides for the most elevated temperature and the greatest decency; that, too, in a space which, while it is sufficient for the emergency, is smaller than that requisite in the other procedures.

In the regenerative system, invented by C. W. and Fr. Siemens, the calefaction is produced by illuminating gas, and requires three distinct agents: a generator, a regenerator, and a chamber, where a given object is submitted to a high temperature, disintegrated and burned. The generator consists of a species of brick oven, in which peat, wood, or charcoal is laid upon a grate and burned. The access of air is limited so as to produce a gas, which is generally a mixture of oxyd of carbon, nitrogen and carburetted hydrogen. This escapes from the generator at a temperature of 150 to 200° C., and then enters the regenerator. The latter is a cube-shaped compartment, with external walls of hard stone, and an interior, crossed by horizontal and vertical walls, arranged in the figure of a grating. This internal masonry becomes highly heated by contact with the combustible gas, which finally enters the combustion-chamber, whence it escapes by a lofty chimney. By the side of this combustion-chamber, a second regenerator is fixed, with grated masonry similar to the first, through which the super-heated air passes to the chimney, and whither the combustible gases are introduced at will, as soon as the first regenerator has attained a red heat. Finally, the air and gases raised to a white heat are introduced into the combustion-chamber, either separately or together, where, by renewing the circulation, they double the heat of the stones, adding to it that of the flame, and occasion an indefinite elevation of temperature. This process is the most expeditious known to me. The opinion of those whom I have consulted in the matter is in accord with my own.

I have had the good fortune to secure the services of two gentlemen who have taken an active interest in this question, and who, with great kindness, have offered me their assistance. M. Steinmann, of Dresden, author of an instructive work on "the Regenerative Process, and the Mode of its Employment," constructed for me, in September, of 1873, an apartment for the dead immediately above the combustion-chamber, from which the bodies are lowered for consumption by the super-heated gases. M. Fr. Siemens, also of Dresden,* improved this new arrangement in December, of 1873, by contriving a more perfect closure of the combustion-chamber. He thought he should also be able to dispense with one of the regenerators. These are not, however, all the improvements which will be supplied; and, were the principle of the process adopted in practice, I should myself propose certain modifications of its details.

Of all modes of cremation, this is the simplest and most satisfactory to the bereaved. Before the friends of the deceased assemble, the body is lowered, with or without coffin, into a confined and empty space, destitute of any other contents. The corpse comes in contact only with air raised to a white heat, whose oxygen at once combines with the atoms of the organic tissue. It burns odorless in this ardent medium, as a candle is consumed without odor in the open air. The ash alone remains, unmingled with any foreign body. The combustion is so perfect that, up to the present time, I have never been able to discover in the return chimney, the pressure of vapor or smoke. Heated air alone escapes.

We propose to publish more fully, at a later date, the results of the experiments which we are together conducting. Thus far, the figures representing the duration of the operation, and the attendant expense, must be accepted with a certain reserve; but one thing is assured, and that is, that the problem which medical science proposes can be solved, if we persevere. The method which I advocate reduces the body with great promptness to an inoffensive residuum, does away with all disagreable impressions upon the bereaved, and accords perfectly with that respect which relatives and friends entertain for the precious remains of those for whom they mourn.

J. N. H.

* Herr Freidrich Siemens has constructed furnaces for combustion of bodies, according to the process described above, which have been tested with satisfactory results since the publication of this article in the *Gazette*. The cost is estimated at $1,250.00, and the time requisite for the cremation of a human body, one hour. On the 3d of June last, two hundred weights of animal tissue were consumed in one hour and a half, and reduced to to white ashes without sound or smell, at an expense of about 75 cents.

---

Dr. E. S. Gaillard has started a new journal entitled *The American Medical Weekly*. *The Northwestern Medical Journal* has been discontinued.

The Council of the British Medical Association has decided in favor of a grant of two hundred pounds to be spent in original researches.

Lactation Late in Life (*Atlanta Med. and Surg. Journal*, July, 1874). —Dr. T. S. Hopkins reports two cases of the return of the functions of the mammary glands after a cessation of seventeen and eighteen years. Both women suckled their grandchildren, one of them being over sixty years of age at the time.

